# Two-level resolution of relative risk of dengue disease in a hyperendemic city of Colombia

**DOI:** 10.1371/journal.pone.0203382

**Published:** 2018-09-11

**Authors:** Aritz Adin, Daniel Adyro Martínez-Bello, Antonio López-Quílez, María Dolores Ugarte

**Affiliations:** 1 Department of Statistics, Computer Science, and Mathematics, Public University of Navarre, Spain; 2 Institute for Advanced Materials (InaMat), Public University of Navarre, Spain; 3 Departament d’Estadística i Investigació Operativa, Facultat de Matemàtiques, Universitat de València, València, Spain; Tulane University, UNITED STATES

## Abstract

Risk maps of dengue disease offer to the public health officers a tool to model disease risk in space and time. We analyzed the geographical distribution of relative incidence risk of dengue disease in a high incidence city from Colombia, and its evolution in time during the period January 2009—December 2015, identifying regional effects at different levels of spatial aggregations. Cases of dengue disease were geocoded and spatially allocated to census sectors, and temporally aggregated by epidemiological periods. The census sectors are nested in administrative divisions defined as communes, configuring two levels of spatial aggregation for the dengue cases. Spatio-temporal models including census sector and commune-level spatially structured random effects were fitted to estimate dengue incidence relative risks using the integrated nested Laplace approximation (INLA) technique. The final selected model included two-level spatial random effects, a global structured temporal random effect, and a census sector-level interaction term. Risk maps by epidemiological period and risk profiles by census sector were generated from the modeling process, showing the transmission dynamics of the disease. All the census sectors in the city displayed high risk at some epidemiological period in the outbreak periods. Relative risk estimation of dengue disease using INLA offered a quick and powerful method for parameter estimation and inference.

## Introduction

Dengue is an arboviral disease caused by a *Flavivirus* belonging to the family *Flaviviridae*, which includes virus transmitted by mosquitoes such as the yellow fever virus, the Zika virus, the West Nile virus, among others. Dengue virus presents four distinct serotypes (DEN-1, DEN-2, DEN-3 and DEN-4) [1] [2], affecting people in tropical and subtropical countries in urban poor areas, suburbs and crowded neighborhoods (World Health Organization [3]). Since 2005, in the world, dengue deaths increased by 48.7% (15.1–90.9), resulting in 18400 deaths (11800–22700) in 2015 [4].

In Latin America, the increasing transmission intensity contributes to growing concerns about other viruses transmitted by *Aedes* mosquitoes, including the Chikungunya and Zika viruses [4], and emergent arboviral diseases such as Mayaro and Oropouche [5]. Racloz *et al*. [6] describe and analyze the epidemiological models attempting to predict dengue outbreaks, concluding that previous studies and modeling efforts have not sufficiently accounted for the spatio-temporal features of dengue disease in the modeling process. Louis *et al*. [7] review tools for surveillance, prevention, and control of dengue focused on mapping dengue risk, finding a high diversity of dengue risk maps representing mainly descriptive and retrospective data. Naish *et al*. [8] review the spatial and spatio-temporal association of dengue disease and environmental, socioeconomic, and climatic factors. They found a diverse frame of statistical methods not integrated to useful public health systems, suggesting the need of combining research efforts to be efficient in dengue surveillance and control.

An specialized branch of disease mapping methods centers in the relative risk estimation on areal data. Relative risk corresponds to the excess (or lack) of disease risk in an area given a local and a global basal risk [9]. Relative risk could be estimated by descriptive or model based approaches. While the first option brings relatively easy and quick results, the great variability inherent to classical risk estimation measures makes necessary to use models to smooth risks using information of spatial and temporal neighbors. Model-based relative risk estimation has been carried out mainly within a hierarchical Bayesian framework in spatial and spatio-temporal disease mapping, with generalized linear mixed models playing a major role. Knorr-Held [10] presented a framework for the spatio-temporal modeling of disease risks for areal data, extending the spatial model of Besag *et al*. [11].

Relative risk estimation of dengue disease has been developed using spatial and spatio-temporal data at several spatial resolutions. For example, spatial modeling of dengue data has been applied to data from Brazil [12] and Colombia [13], while spatio-temporal dengue data have been analyzed using relative risk models in Brazil [14–16], Ecuador [17], Thailand [18], Colombia [19] [20], and Indonesia [21]. However, most of these analyses did not fully explore the space-time interaction effect model framework. Additive models were considered by Lowe *et al*. [14–16, 18] and Stewart-Ibarra *et al*. [17]. A simple spatio-temporal unstructured interaction effect model was used by Wijayanti *et al*. [21] while Martínez-Bello *et al*. [20] applied models of dengue relative risks with full space-time interaction terms.

Colombia is an endemic country for dengue disease. This can be attributed to high density of people living in cities and towns, with environmental and climatic conditions favouring the dengue vector development [22]. The city of Bucaramanga is one of the Colombian cities with the highest annual incidence of dengue disease in the period 2009-2015, being one of the cities where dengue vaccines have been tested [23] [24] with some criticism from some Colombian researchers [25]. While Colombia presented an incidence rate of 624 cases per 100,000 inhabitants in 2010, Bucaramanga reported an incidence rate of 1322.1 per 100,000 inhabitants. Data on incident cases of dengue disease (dengue and severe dengue) were obtained from the SIVIGILA (Colombian public health surveillance system) for the urban area of Bucaramanga for the period from January 2009 to December 2015, and a battery of spatio-temporal models including two-level spatial effects were fitted [26] [27]. Ugarte *et al*. [28, 29] also used spatio-temporal models with two-level spatial effects for analysing the evolution of young people’s brain cancer mortality in Spanish provinces, and for studying the temporal trends of brain cancer incidence in the municipalities of two regions located in the North of Spain, respectively.

The aim of the study is to analyze the geographical distribution of relative incidence risk of dengue disease in the city of Bucaramanga and its evolution in time during the period January 2009—December 2015, identifying regional effects at different levels of spatial aggregations.

## Materials and methods

### Cases of dengue disease from Bucaramanga, Colombia

Dengue cases from the city of Bucaramanga were geocoded and allocated to one of the 94 census sectors. A census sector is a cartographic unit obtained from the aggregation of census sections which at the same time are the aggregation of census blocks. A census block is “*a lot of built or unbuilt land bounded by vehicular or pedestrian traffic roads of a public nature*”, a census section is “*a cartographic bounded urban division approximately equal to 20 contiguous census blocks and belonging to a urban sector*”, while a census sector is a “*census cartographic division at urban level, generally equivalent to a neighbor (in the principal cities), comprising between 1 to 9 census sections*” (definitions adapted from [30]). The census sectors are nested in communes. The commune is an administrative division in the municipality, representing census sectors sharing similar geographical and physical characteristics. The city of Bucaramanga covers an urban area of 27 km^2^, with a population of 527,913 people (projection 2016) living in 94 census sectors nested in 17 communes. The city is located at 959 m above sea level with the coordinates 7°7′07′′ N—73°06′58′′ W.

The inclusion of the commune as a second level of aggregation is justified by two reasons. First, a great burden of the analysis and intervention of the notification diseases at municipal level is undertaken by the health authorities employing the geographical division comprised by the communes. However, the key health event corresponding to the dengue case occurs at house or household level. For the cases at hand, working at house or household level is challenging from the computational side, then the cases were aggregated at census sector for the sake to represent the dengue risk at small spatial scale. Secondly, as expressed above, the commune corresponds to a physical division of the city by neighborhoods and city blocks delimited by clear spatial divisions. If we include the vector biology of the disease (the mosquito *Aedes aegypti*) within the risk estimation of dengue, we could think that the vector is confined to small areas sharing special conditions for the mosquito development, which is accounted with a second level of aggregation such as the commune.

The geocoding process followed the next protocol: dengue cases data were obtained from the surveillance system of public health (SIVIGILA) for the period January 2009 to December 2015. The SIVIGILA database is an online system allowing the Colombian health institutions to register the diseases of obligatory notification. The dengue data included address, sex, age, and an identification code that anonymizes the name and personal identity of the case to the geocoder. The geocoding process started with a database checked for duplicates of 39,775 records corresponding to the notified dengue cases from health institutions in Bucaramanga. Only the records with address of residence belonging to Bucaramanga were considered, discarding cases without address, with rural address or wrong addresses. An R software [31] script sent batches of addresses to the web geocoding service of ArcGIS server. The web server returned JSON files, which were checked and accepted, or revised for a new geocoding cycle. At the end of the process, we successfully geocoded to the urban area of Bucaramanga a total of 25,365 cases. Then, the coordinates obtained from the geocoding process belonging to every dengue case were allocated to census sectors using the cartography generated by the National Geostatistical Framework, 2005 [30]. In addition, the cases were temporally aggregated in epidemiological periods, composed by four epidemiological weeks, for the entire study period. The epidemiological period is the common time measure employed by the health offices in South and Central America, with a total of 91 epidemiological periods between January 2009 and December 2015 (13 epidemiological periods by year, and 7 epidemiological years).

We obtained disaggregated data by census sector, sex, and five-years age groups from the Colombian Census 2005, and calculated a cumulative crude incidence rate according to these variables. We computed cumulative expected dengue cases per area (census sectors and communes) and the seven-years period as the product of the cumulative crude incidence rate and the population at risk by age-groups and sex in every census sector and commune. Then, we added the cumulative expected cases per census sector and commune by age-group and sex, obtaining the cumulative expected cases per area. Finally, the cumulative expected dengue cases were divided by the number of epidemiological periods to obtain expected cases per area and epidemiological period.

### Two-level spatially structured models in space-time disease mapping

Let us assume that the city of Bucaramanga is divided into *n* census sectors labeled as *i* = 1, …, *n*, that are nested into *m* communes labeled as *j* = 1, …, *m*. For each census sector *i*, data are available for different epidemiological periods labeled by *t* = 1, …, *T*. Let *O*_*it*_, *e*_*it*_, and *r*_*it*_ denote the number of observed dengue cases, the number of expected dengue cases, and the relative risk of dengue disease for census sector *i* and epidemiological period *t*, respectively. Then, conditional on the relative risk, the number of counts is assumed to be Poisson distributed with mean *μ*_*it*_ = *e*_*it*_*r*_*it*_, that is,

$$\begin{array}{c} O_{it}|r_{it}∼\text{Poisson}(\mu_{it}=e_{it}r_{it}) \end{array}$$ (1)

$$\begin{array}{c} \text{log}\;\mu_{it}=\text{log}\;e_{it}+\text{log}\;r_{it}. \end{array}$$ (2)

Depending on the specification of log *r*_*it*_ several models could be defined. Most of the research in space-time disease mapping is based on conditional autoregressive (CAR) priors for both spatial and temporal effects (Knorr-Held [10]). Extensions of these models were proposed by Ugarte *et al*. [27] for analyzing small area data that are naturally grouped into larger regions. The models include two-level of spatially structured random effects, identifying regional effects and modeling space-time interactions at different levels of spatial aggregations. In what follow, we briefly describe some of these models.

First, a model with census sector level space-time interaction has been considered (hereafter *TL-Model A*), where the log-risk is modeled as

$$\begin{array}{c} \text{log}\;r_{it}=\eta +\xi_{i}+\psi_{j(i)}+\gamma_{t}+\delta_{it}, \end{array}$$ (3)

where *j*(*i*) denotes that census sector *i* belongs to the commune *j* = 1, …, *m*. Here *η* is an intercept representing an overall level of risk, *ξ*_*i*_ and *ψ*_*j*(*i*)_ are census sector and commune level spatially structured random effects respectively, *γ*_*t*_ is a temporally structured random effect, and *δ*_*it*_ is the space-time interaction effect that models the dependence between the census sectors and the epidemiological periods. If the interaction term is dropped, an additive model is obtained. A Leroux *et al*. [32] CAR (LCAR) prior distribution is given to both spatial random effects, that is,

$$\begin{array}{c} \xi ={\left(\xi_{1},\ldots ,\xi_{n}\right)}^{{}^{\prime }}∼N(0,{\left[\tau_{\xi }\left(\lambda_{\xi }R_{\xi }+\left(1-\lambda_{\xi }\right)I_{n}\right)\right]}^{-1}), \end{array}$$ (4)

$$\begin{array}{c} \psi ={\left(\psi_{1},\ldots ,\psi_{m}\right)}^{{}^{\prime }}∼N(0,{\left[\tau_{\psi }\left(\lambda_{\psi }R_{\psi }+\left(1-\lambda_{\psi }\right)I_{m}\right)\right]}^{-1}), \end{array}$$ (5)

where *τ*_*ξ*_ and *τ*_*ψ*_ are precision parameters, λ_*ξ*_ and λ_*ψ*_ are spatial smoothing parameters taking values between 0 and 1, **I**_*n*_ and **I**_*m*_ are identity matrices of dimension *n* × *n* and *m* × *m* respectively, **R**_*ξ*_ is the *n* × *n* neighborhood matrix of the census sectors, and **R**_*ψ*_ is the *m* × *m* neighborhood matrix of the communes. Note that spatial independence is assumed when the spatial smoothing parameters are equal to zero, while intrinsic CAR prior distributions are considered when these parameters are equal to one. A first order random walk (RW1) prior distribution is given for the temporally structured random effect, that is,

$$\begin{array}{c} \gamma ={\left(\gamma_{1},\ldots ,\gamma_{T}\right)}^{{}^{\prime }}∼N\left(0,{\left[\tau_{\gamma }R_{\gamma }\right]}^{-}\right). \end{array}$$ (6)

Here *τ*_*γ*_ is a precision parameter and **R**_*δ*_ is the *T* × *T* structure matrix of a RW1.

Finally, the following prior distribution is assumed for the space-time interaction random effect *δ* = (*δ*_11_,…,*δ*_1T_,…,*δ*_*n*1_,…,*δ_nT_*)′

$$\begin{array}{c} \delta ∼N(0,{\left[\tau_{\delta }R_{\delta }\right]}^{-}). \end{array}$$ (7)

Here *τ*_*δ*_ is a precision parameter and **R**_*δ*_ is the *nT* × *nT* matrix obtained as the Kronecker product of the corresponding spatial and temporal structure matrices. Note that a commune level interaction effect can be also considered in the model of Eq (3), modeling the log-risks as (hereafter *TL-Model B*)

$$\text{log}\;r_{it}=\eta +\xi_{i}+\psi_{j(t)}+\gamma_{t}+\delta_{j(i)t.}$$ (8)

As proposed by Knorr-Held [10], four types of space-time interactions can be defined for TL-Model A and TL-Model B (see Table 1).

**Table 1:** Specification for the different types of space-time interactions.

Interaction		Structure	
Type	R_*δ*_	Spatial	Temporal
Two Level-Model A			
I	**I***_n_* ⊗ **I***_T_*	−	−
II	**I***_n_* ⊗ **R**_*γ*_	−	✓
III	**R***_ξ_* ⊗ **I**_*T*_	✓	−
IV	**R***_ξ_* ⊗ **R**_*γ*_	✓	✓
Two Level-Model B			
I	**I***_m_* ⊗ **I***_T_*	−	−
II	**I***_m_* ⊗ **I***_γ_*	−	✓
III	**R***_ψ_* ⊗ **I**_*T*_	✓	−
IV	**R***_ψ_* ⊗ **I**_*γ*_	✓	✓

A sensible modification of these models is to account for spatial variability only among those census sectors belonging to the same commune. In this case, the census sector level random effects are distributed as $\xi^{*}∼N\left(0,{\left[\tau_{\xi }\left(\lambda_{\xi }R_{\xi }^{*}+\left(1-\lambda_{\xi }\right)I_{n}\right)\right]}^{-1}\right)$, where $R_{\xi }^{*}=\text{blockdiag}\left(R_{\xi_{1}},\ldots ,R_{\xi_{m}}\right)$ is a block-diagonal matrix and $R_{\xi_{j}}$ is the neighborhood matrix of census sectors within the *j*th commune. Both census sector or commune level space-time interactions can be considered, defining the following models

$$\begin{array}{c} \begin{array}{cc} TL-Model\;C: & \;\text{log}\;r_{it}=\eta +\xi_{i}^{*}+\psi_{j(i)}+\gamma_{t}+\delta_{it}^{*},\\ TL-Model\;D: & \;\text{log}\;r_{it}=\eta +\xi_{i}^{*}+\psi_{j(i)}+\gamma_{t}+\delta_{j(i)t}. \end{array} \end{array}$$ (9)

Again, four different types of space-time interaction can be defined for the models of Eq (9), obtained as the Kronecker product of the corresponding spatial and temporal structure matrices.

### Model inference and estimation

Different spatio-temporal models of relative risk described above were fitted using the integrated nested Laplace approximation (INLA) technique, an approximate method for Bayesian inference for latent Gaussian models developed by Rue *et al*. [33]. INLA provides reliable results in short computational time when the precision matrices of the random effects are sparse, allowing to make Bayesian inference without running long and complex Markov chain Monte Carlo (MCMC) algorithms. Spatio-temporal models of relative risk using LCAR priors for the spatially structured effects have been fitted by Ugarte et al [26] using INLA, while two-level spatio-temporal models have been formulated and implemented in INLA by Ugarte et al. [27]. This technique can be used in the free statistical software R through the R-INLA package. Appropriate identifiability constraints have been considered for each model, which are derived by re-parameterizing the random effects using the spectral decomposition of their precision matrices (see Goicoa *et al*. [34]). Non-informative prior distributions were assigned to the model hyperparameters as follows

$$\begin{array}{c} \eta ∼\text{Normal}(0,1000), \end{array}$$ (10)

$$\begin{array}{c} \lambda_{\xi },\lambda_{\psi }∼\text{Uniform}(0,1), \end{array}$$ (11)

$$\begin{array}{c} \frac{1}{\sqrt{\tau_{\xi }}},\frac{1}{\sqrt{\tau_{\psi }}},\frac{1}{\sqrt{\tau_{\gamma }}},\frac{1}{\sqrt{\tau_{\delta }}}∼\text{Uniform}(0,\infty ).\; \end{array}$$ (12)

Some model selection criteria were considered to compare the different models in terms of model fitting and complexity. The deviance information criterion (DIC) (Spiegelhalter *et al*. [35]) is the most commonly used measure of model fit based on the deviance for Bayesian models, which is computed as the sum of the posterior mean of the deviance $\bar{D}$ (a measure of goodness of fit) and the number of effective parameters *p*_*D*_ (a measure of model complexity). Although the use of the DIC has been widespread during the last years, it has been criticized by several authors in the literature. It is recognized that the DIC values may underpenalize complex models containing random effects in disease mapping, so the corrected version of the DIC proposed by Plummer [36] was also considered in this paper. It is also known that the DIC can produce negative estimates of the effective number of parameters in a model. Some authors recommend the use of the Watanabe-Akaike information criterion (WAIC) (Watanabe [37]) instead of the DIC (see for example, Gelman *et al*. [38]; Vehtari *et al*. [39]). The WAIC criterion was also computed here. Finally, we provide the cross-validate logarithmic score (LS) (Gneiting and Raftery [40]) as a criterion based on the model posterior predictive distribution.

## Results

### Summary statistics

**Fig 1:**
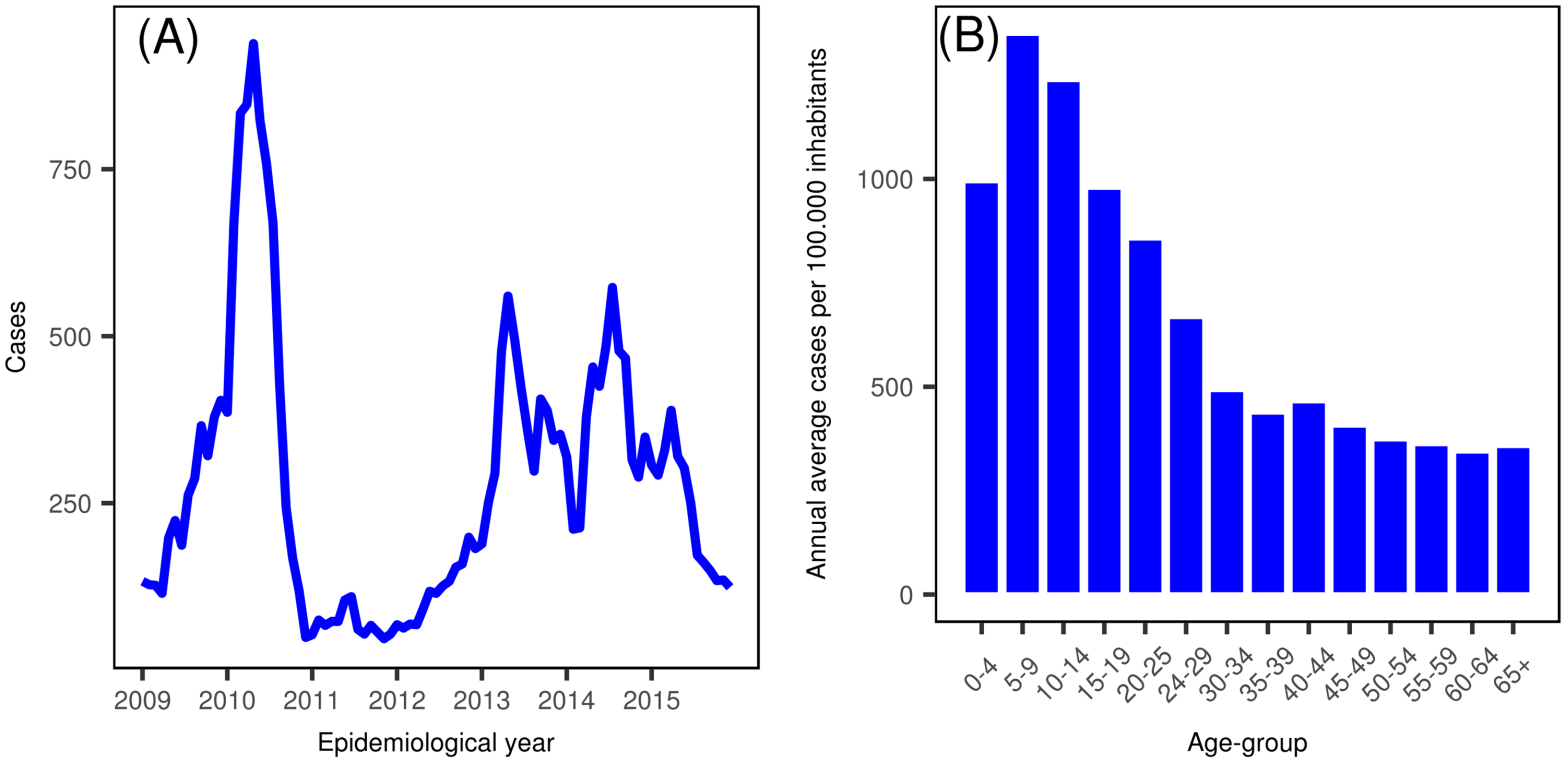
Descriptive analysis of dengue disease cases in the city of Bucaramanga, Colombia. (A) Cases by epidemiological period. (B) Annual average cases per 100.000 inhabitants by age-groups.

A total of 25,365 dengue cases were successfully geocoded to the city area of Bucaramanga. As shown in Fig 1A, three main outbreaks were experienced in the city during the period January 2009 to December 2015: in the first semester of 2010 with around 940 cases, and in the first semester of 2013 and 2014 presenting near to 550 cases each. Fig 1B shows the age-groups [5–9] and [10–14] years presenting the highest annual average cumulative incidence of dengue disease for the study period (1,349 and 1,238 cases by 100,000 inhabitants, respectively). The maximum number of dengue cases per census sector and commune were 47 and 97 cases respectively.

**Fig 2:**
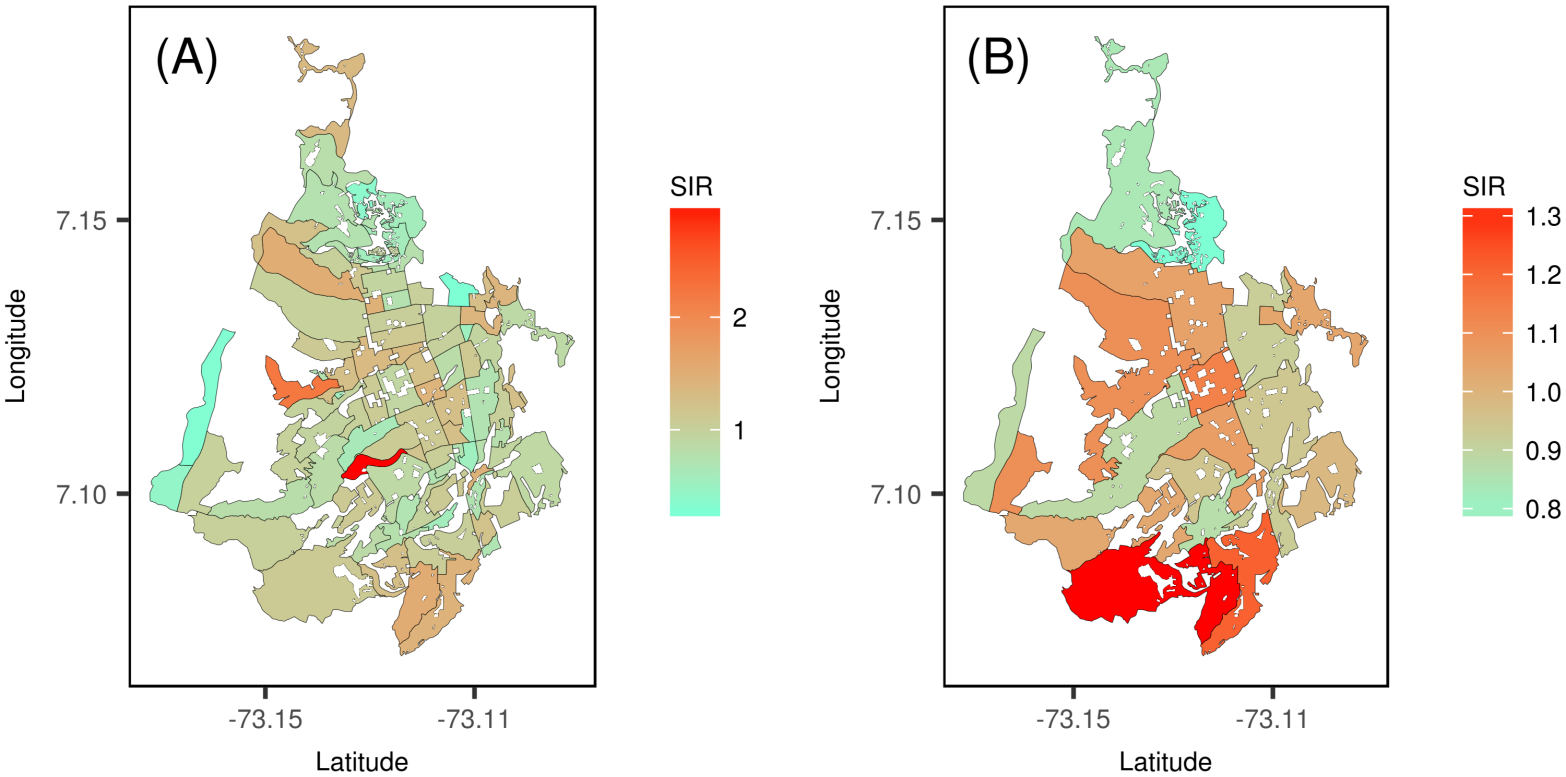
Cumulative standardized incidence rates (SIRs) of dengue disease by communes and census sectors. (A) SIR of dengue cases by census sector. (B) SIR of dengue cases by commune.

Fig 2 provides the cumulative standardized incidence rate (SIR) of dengue disease in the 293 census sectors and 94 communes for the 7-year time period (2009–2015). The cumulative SIR of dengue is an indirect method of adjustment for age and sex, acting as a measure to compare dengue cases in each area and time point with the whole city during the study period. The cumulative SIR per census sector (Fig 2A) shows a diffuse incidence pattern with a few high incidence census sectors to the west of the city, while the cumulative SIR per commune (Fig 2B) reveals high incidence to the south and central communes of the city.

### Results from the selected model

Table 2 shows the results from fitting the models described above with R-INLA using the simplified Laplace approximation strategy. In general, the models with census sector level interaction effect are better than those considering a commune level interaction effect. Nevertheless, from a computational point of view, the latter models are much faster because the space-time precision matrix (**R**_*δ*_) has lower dimension and less identifiability constraints are needed. In addition, according to the different model selection criteria, the model with the usual spatial neighborhood structure performs better than TL-Models C and D that incorporate a more complex neighborhood structure between areas. As reported in Table 2, TL-Model A with completely structured (Type IV) interaction random effect shows the lowest values for all the model selection criteria considered here (almost 80 units less than TL-Model A and C with Type II interaction effect in terms of DIC and WAIC, and 150 units less in terms of corrected DIC).

**Table 2:** Model selection criteria for the best fitted models in INLA: Mean deviance ($\bar{D}$), number of effective parameters (*p*_*D*_), deviance information criterion (DIC), corrected DIC (DICc), Watanabe-Akaike information criterion (WAIC) and logarithmic score (LS).

	*TL-Model A*: log *r*_*it*_ = *η* + *ξ*_*i*_ + *ψ*_*j*(*i*)_+ *γ*_*t*_ + *δ*_*it*_					
Space-time interaction	$\bar{D}$	p_*D*_	DIC	DICc	WAIC	LS
Additive model	30256.0	162.8	30418.8	30423.1	30552.6	15276.8
Type I	26300.3	2282.4	28582.7	30711.9	28604.0	14816.0
Type II	26792.8	1266.2	28059.0	28406.8	28251.7	14196.6
Type III	26762.0	1733.1	28495.1	29652.5	28709.7	14706.5
Type IV	26885.7	1095.4	**27981.1**	**28256.1**	**28167.3**	**14138.8**
	*TL-Model B*: log *r*_*it*_ = *η* + *ξ*_*i*_ + *ψ*_*j*(*i*)_ + *γ*_*t*_ + *δ*_*j*(*i*)*t*_					
Space-time interaction	$\bar{D}$	p_*D*_	DIC	DICc	WAIC	LS
Type I	28451.1	851.1	29302.2	29521.0	29713.3	14917.3
Type II	28526.7	578.3	29105.0	29186.1	29388.4	14711.6
Type III	28560.7	821.9	29382.5	29587.5	29794.5	14955.6
Type IV	28572.1	575.1	29147.2	29231.1	29431.6	14734.1
	*TL-Model C*: $\text{log}\;r_{it}=\eta +\xi_{i}^{*}+\psi_{j(i)}+\gamma_{t}+\delta_{it}^{*}$					
Space-time interaction	$\bar{D}$	p_*D*_	DIC	DICc	WAIC	LS
Additive model	30256.7	162.9	30419.6	30424.0	30553.3	15277.1
Type I	26307.5	2276.7	28584.2	30699.1	28608.9	14814.8
Type II	26789.2	1269.2	28058.3	28407.9	28249.9	14196.1
Type III	28210.7	1467.5	29678.3	30453.5	30263.7	15844.3
Type IV	28255.5	899.1	29154.6	29328.6	29514.9	14790.5
	*TL-Model D*: $\text{log}\;r_{it}=\eta +\xi_{i}^{*}+\psi_{j(i)}+\gamma_{t}+\delta_{j(i)t}$					
Space-time interaction	$\bar{D}$	p_*D*_	DIC	DICc	WAIC	LS
Type I	28451.3	851.8	29303.1	29522.0	29714.2	14917.8
Type II	28527.7	578.4	29106.1	29187.2	29389.5	14712.2
Type III	28561.9	821.6	29383.5	29588.1	29795.3	14955.9
Type IV	28573.2	575.1	29148.3	29232.1	29432.7	14734.7

Finally, Table 2 shows that the number of effective parameters decreases for Type II and IV interaction models in comparison with Type I and III. This might seem counterintuitive since a Type IV interaction model is more complex in terms of the covariance structure induced for the space-time neighboring points. However, we note that the number of effective parameters is also an indicator of the degree of smoothness induced by the model. As the random effects of the model induce more smoothness, i.e., as the shrinkage towards zero (the mean) is stronger, the more we move away from the saturated model, and therefore the model is less complex. This seems the be the reason why models with Type I or III interaction random effects, that do not induce smoothing effects between temporal neighbors, shows higher values of *p*_*D*_.

Table 3 shows the summary statistics for the precision parameters from the selected model. The posterior mean of the spatial smoothing parameter of the LCAR prior distribution for the census sector random effect (λ_*ξ*_) is 0.283, which is interpreted as small spatial dependence between these areas. For the commune random effect, the posterior mean of the spatial smoothing parameter (λ_*ψ*_) is 0.448, indicating a moderate spatial dependence between communes in the same study period.

**Table 3:** Summary statistics for the precision parameters of the TL-Model A with type IV interaction effect for the relative risk of the Dengue, Jan 2009–Dec 2015.

Parameter	Mean	SD	Q 0.025	Q 0.5	Q 0.975
*τ*_*ξ*_	6.08	1.93	3.31	5.73	10.81
λ_*ξ*_	0.28	0.156	0.058	0.27	0.60
*τ*_*ψ*_	58.82	78.04	6.72	35.59	253.68
λ_*ψ*_	0.44	0.24	0.06	0.43	0.89
*τ*_*γ*_	19.20	3.23	13.51	18.99	26.16
*τ*_*δ*_	14.19	1.18	12.04	14.14	16.66

**Fig 3:**
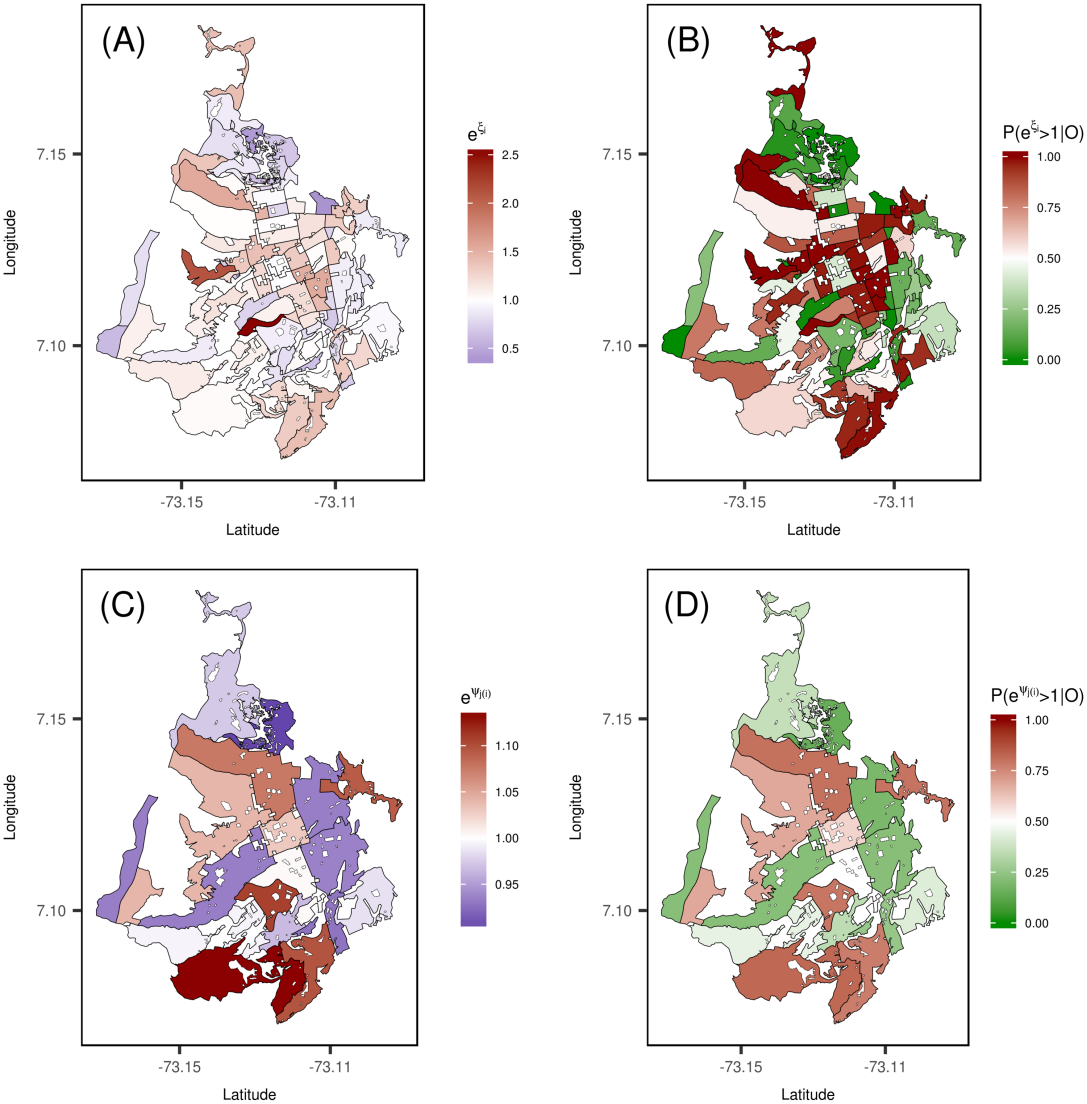
Posterior mean estimates of spatial random effects at both census sector and commune-level, and posterior exceedance probability of being greater than one. (A) Map of census sector level spatial incidence risk pattern exp(*ξ*_*i*_). (B) Posterior probability distribution P(exp(*ξ_i_*) >1|**O**). (C) Map of commune level spatial incidence risk pattern exp(*ψ*_*j*(*i*)_). (D) Posterior probability distribution P(exp(*ψ_j(i)_*) > 1|**O**).

By fitting spatio-temporal models with two-level of spatial random effects, we provide a tool to establish the association between the commune and the census sector with the relative risk of dengue disease, accounting for those geographical factors specific to the area covered by the commune. Fig 3 shows the maps for the census sector and commune level spatial incidence risk patterns (constant during the whole period) derived from the selected model. These spatial patterns, can be interpreted as the specific contribution of the area to the increase/decrease of the relative risks *r*_*it*_. Fig 3A exposes some of the census sector located in the central areas of the city showing a large mean spatially structured pattern. The probability of the census sector spatial effects being greater than one is represented in Fig 3B. At commune level, a large spatial incidence patterns is observed in the southern and western communes of the city (Fig 3C), which is better inferred by the posterior exceedance probabilities P(exp(*ψ_j(i)_*) > 1|**O**) represented in Fig 3D.

Including two-level random effects in the model allow us to identify those census sectors and/or communes that have a significant effect on the relative risk. For example, the commune located further north in the city of Bucaramanga it is not a high risk area, but some of its census sectors show a high probability that the spatial effect is significantly higher in comparison with the whole of the sectors (see Fig 3). In this way, we are able to identify those high/low risk areas that show behaviors associated to both levels of spatial aggregation.

**Fig 4:**
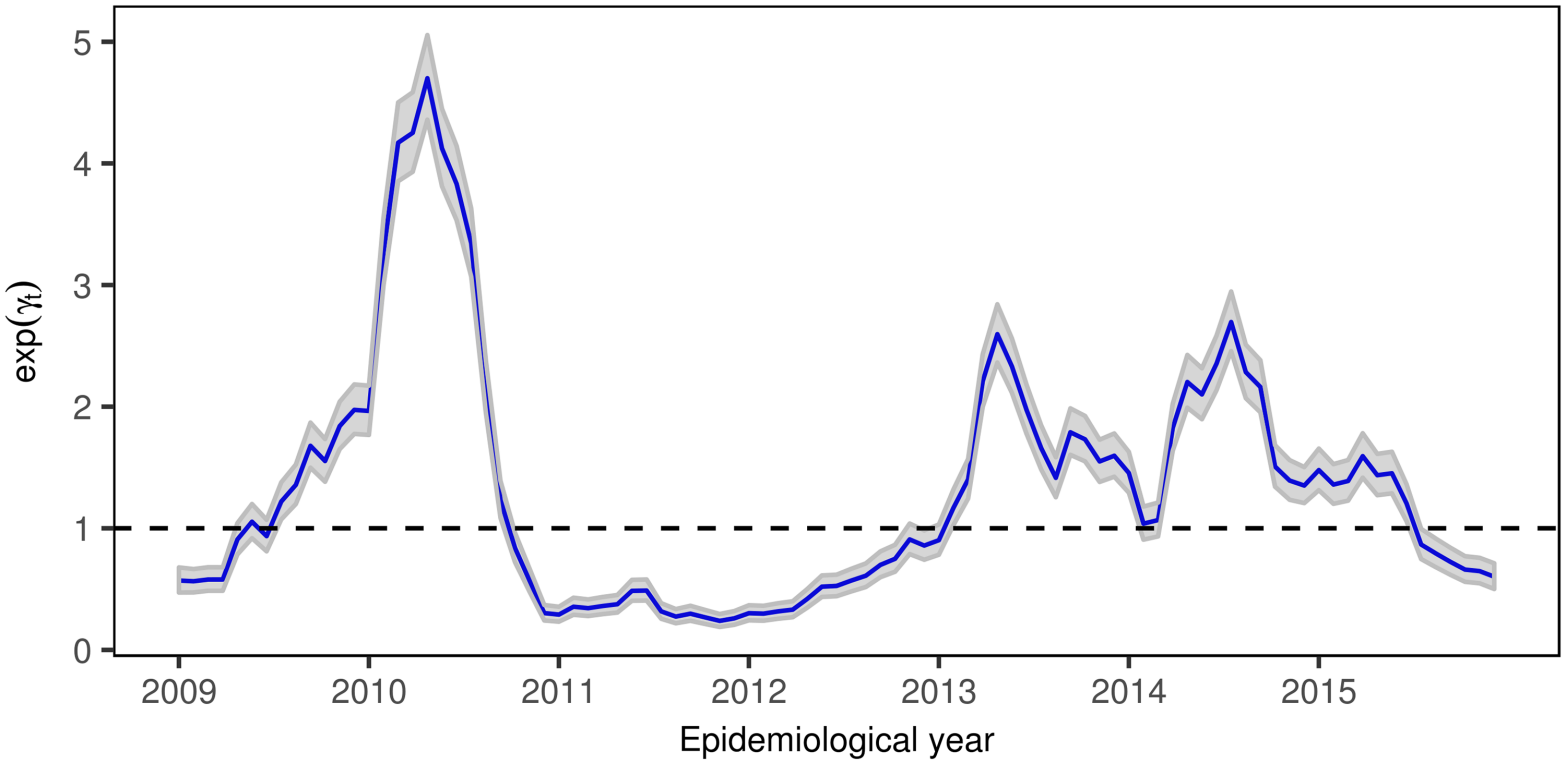
Overall temporal trend of dengue disease incidence relative risk by epidemiological period, exp(*γ*_*t*_), and 95% credibility intervals.

The posterior mean temporal trend and 95% credible intervals by epidemiological period (common to all areas) is shown in Fig 4, recovering the high risk pattern of dengue disease in the first semesters of 2010, 2013, 2014, and 2015.

**Fig 5:**
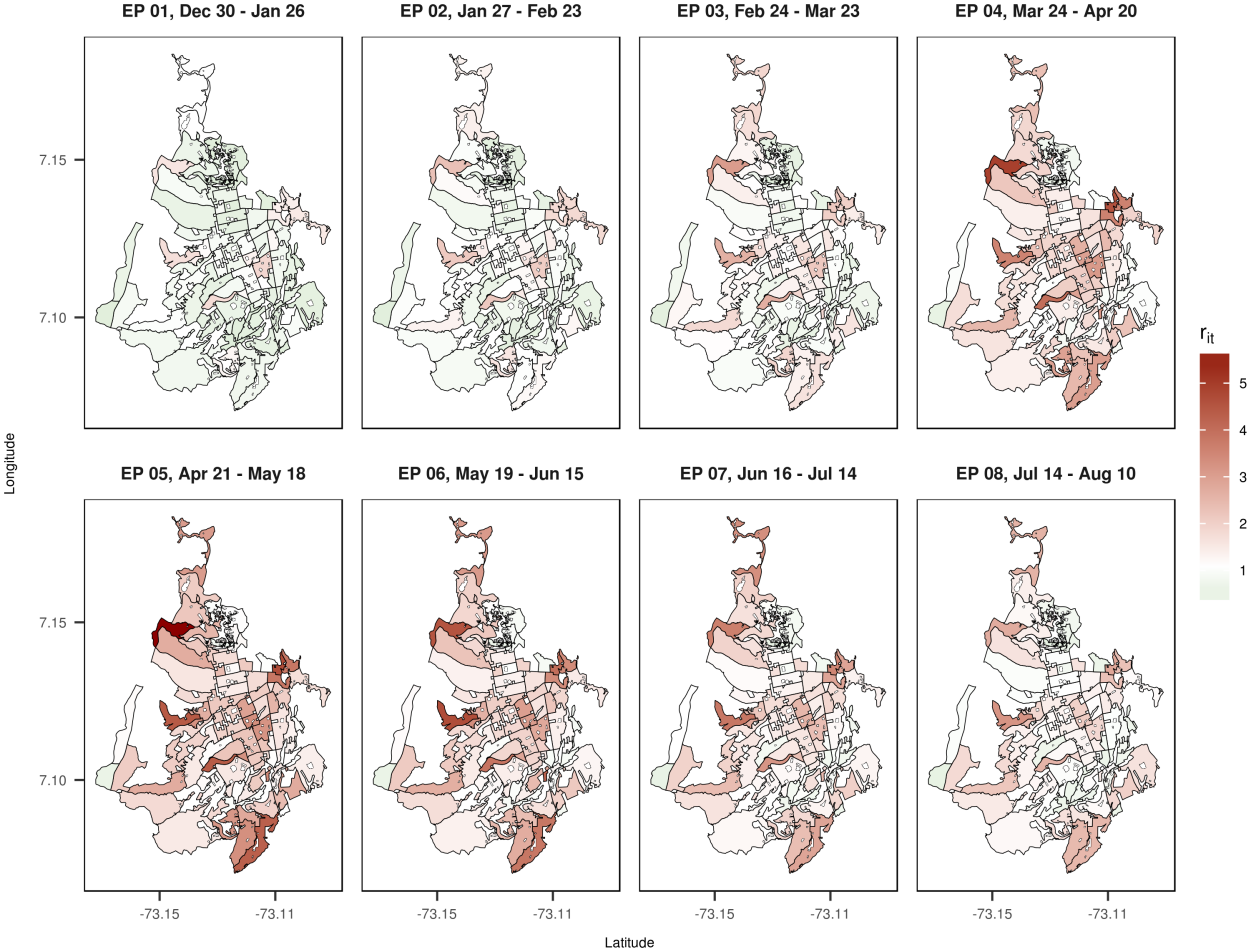
Maps with the estimated posterior mean values of the relative risk *r*_*it*_ of dengue disease by census sector for the epidemiological periods 1 to 8 of 2013.

**Fig 6:**
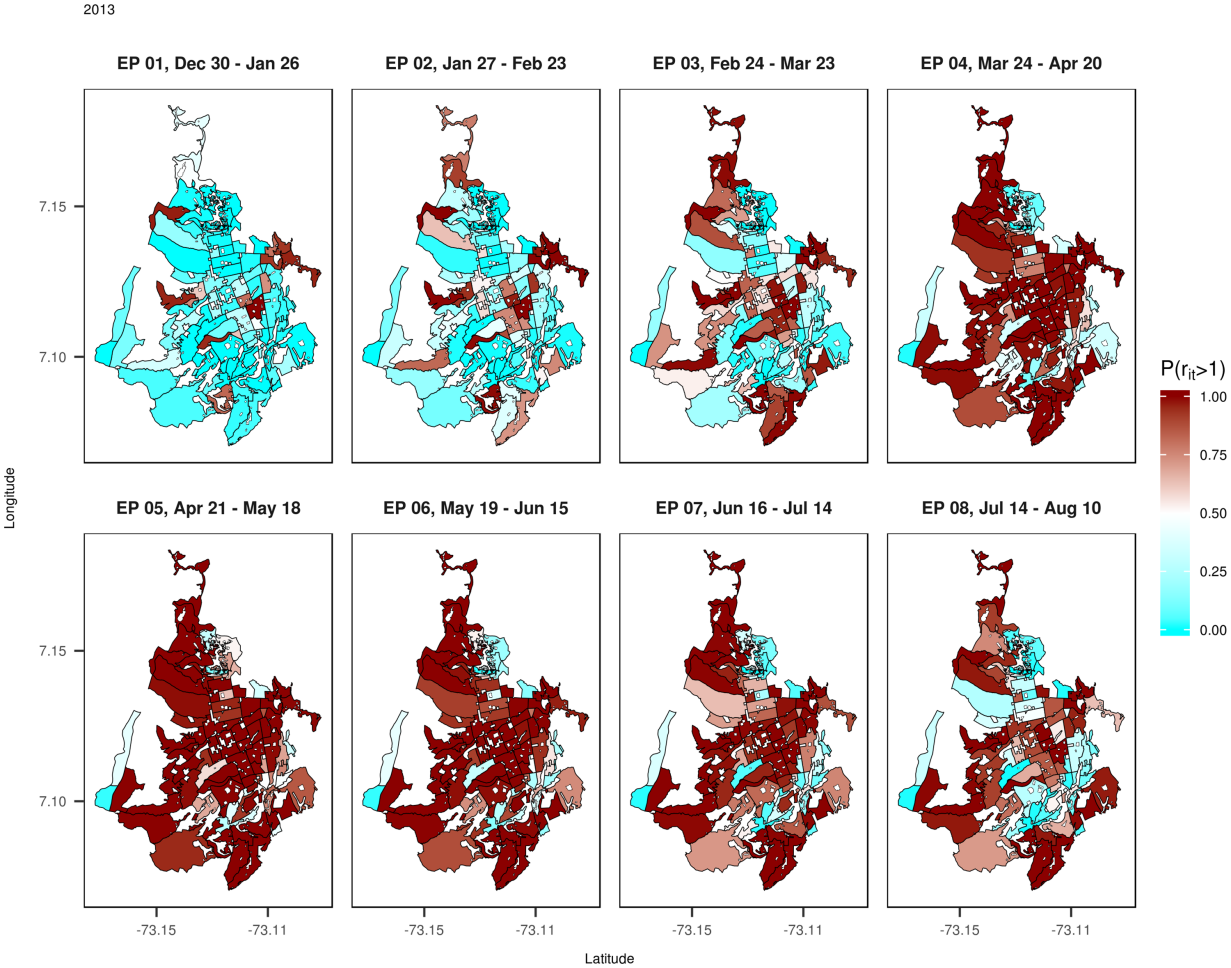
Maps of the posterior probability distribution P(*r_it_* > 1|O) of dengue disease by census sector for the epidemiological periods 1 to 8 of 2013.

Mapping the relative risk estimates of dengue disease is one of the main outputs from the modeling process. We have chosen the epidemiological periods 1 to 8 from the year 2013 to display the estimated posterior mean values of the relative risk of dengue disease (Fig 5). Using the relative risk implies that the one is the basal risk. The maps in Fig 5 show that in 2013, the EP 1 and 2 present a low overall relative risk in most of the census sectors, but afterwards, the relative risk spread from the center of the city in EP 3 and 4 to the rest of census sectors in EP 5 and 6, and finally decreasing slightly in the EP 7 and 8. To detect the areas with high relative incidence risk, maps of the posterior exceedance probabilities P(*r_it_* > 1|**O**) by census sector and epidemiological period have been represented in Fig 6. This posterior probability distribution provides a kind of Bayesian p-value, which it could be used to detect or highlight high risk areas based on the definition of a cut point by the analyst.

**Fig 7:**
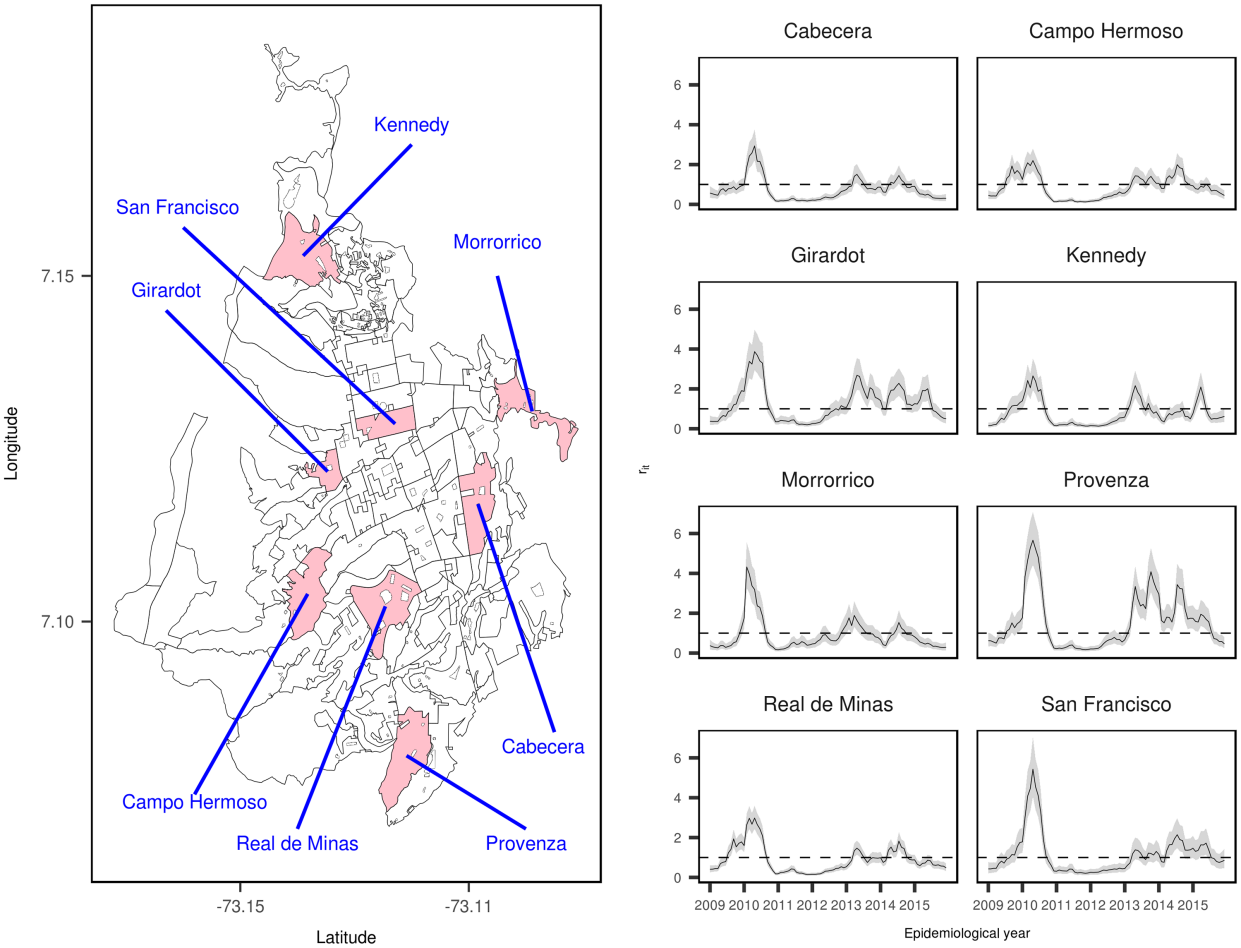
Map of selected census sector to display relative risk of dengue disease for the period Jan 2009–Dec 2015 (left panel), and specific temporal evolution of the posterior mean estimates of relative risk and 95% credible intervals (right panel).

Finally, we have selected eight census sector distributed across the city to plot their specific temporal evolution of dengue incidence risk during the period Jan 2009–Dec 2015, and the posterior mean values of the estimated relative risks and 95% credible intervals by epidemiological period (Fig 7). Four census sectors correspond to the central areas of the city (central east area: *Cabecera* sector; central north area: *San Francisco* sector; central south area: *Real de Minas* sector; and central west area: *Campohermoso* sector), and four census sectors from the east (*Morrorico* sector), north (*Kennedy* sector), south (*Provenza* sector) and west (*Girardot* sector) areas of the city. Although the temporal evolution of relative risks are similar to the main temporal pattern represented in (Fig 4), subtle differences are revealed between census sectors. The main outbreak of dengue cases observed in the city of Bucaramanga (first semester of 2010) did not equally affect to all areas, observing significantly higher spikes in *Provenza*, *San Francisco*, and *Morrorrico* sectors. In addition, quite different relative risk evolutions are observed during the period 2013 to 2015. Sectors located in the central areas of the city show much more moderate risks during the last years of the analyzed period than the areas located in the suburbs of the city, where significantly high relative risks are observed in *Provenza* and *Girardot* sectors.

## Discussion

In this work, spatio-temporal disease mapping models are applied to dengue incidence data in the city of Bucaramanga (Colombia). Dengue cases are spatially aggregated at two different administrative levels, census sectors and communes, and temporally aggregated in epidemiological period. This is the first report where models with two-level of spatially structured random effects have been used to estimate dengue disease incidence relative risks in small areas. These models permit to identify regional effects at each level of spatial aggregation, considering space-time interaction effects at census sector or commune level. We found for the particular data at hand that the best model to report the results includes spatial random effects for both census sector and commune levels, a temporally structured random effect for the epidemiological periods, and a completely structured interaction term over the census sectors (TL-Model A with type IV interaction). Different model selection criteria have been used to compare the behaviour of the fitted models, such as the deviance information criterion, the Watanabe-Akaike information criterion or the cross-validate logarithmic score.

As pointed out by one reviewer, TL-Model C with type II interactions (temporally structured trends that are spatially unstructured) is the closest model to the one selected (TL-Model A), based on the similar characteristics and the results of the model selection statistics. The difference between TL-Model A and C is that model A uses a proximity matrix which considers the neighboring structure of adjacent census sectors independently of the commune borders, while model C ignores the neighboring structure of census sectors between communes, so the connection of census sectors nested in every commune are bounded by the commune borders. Although TL-Model C is computationally faster than TL-Model A, all the model selection criteria pointed out to TL-Model A with type IV interaction effects as the best model for analyzing the dengue data. Indeed, this final selected model seems to have a more sensible interpretation than TL-Model C as it seems natural to expect that temporal trends of dengue risk in neighbouring census sectors is similar ignoring the commune borders.

The selected model implies that the risk of dengue disease on every census sector is highly associated to the neighboring census sectors in space and time, where the commune effect plays an important role in the dynamics of the transmission of dengue disease across the city, together with the risk in the census sectors directly connected to the census sectors in adjacent communes. For the comprehension of the dengue transmission in the city, selecting the TL-Model A means that as much the adjacency between communes as the adjacency between census sectors in the communes having adjacent bounds are associated to those census sectors displaying high-risk of dengue disease.

The model’s output allows creating risk maps by epidemiological period, and risk profiles by census sector through the study period. Inspection of the risk maps permits to detect high/low risk areas in comparison with all the census sectors of the city across the period January to December 2015. Our analysis shows differences in dengue incidence risk between sectors situated in central areas of the city compared to sectors located in the suburbs of the city.

The present analysis extends the results shown by Martínez-Bello *et al*. [41], where the time series of dengue disease in the city of Bucaramanga were analyzed in a weekly basis, including meteorological covariates. The overall and sector-specific temporal trends of dengue disease obtained from the spatio-temporal model allow the comparison of the longitudinal profiles of dengue risk per sector.

The main novelty in the present work is the inclusion of the commune effect as a second level of spatial aggregation in the modeling process. The primary benefit of considering models with two-level spatial random effects is that they provide key information to the public health policy-makers, such as the geographical distribution of dengue disease relative risks by census sector, and the contribution of the communes to the increase/decrease of these risks. Models with a single level of spatial dependence were considered in Martinez *et al*. [20] to analyze dengue incidence data in the city of Bucaramanga at smaller spatial aggregation units (census sections), including covariates obtained from satellite data. However, particular attention must be paid on solving identifiability issues in disease mapping models when covariates are included, because ignoring the spatial or temporal correlation between covariates and the random effects can lead to misleading results due to confounding issues (see for example, Reich *et al*. [42]; Hodges and Reich [43]; Goicoa *et al*. [34]). It is a matter of further research not only how to deal with covariates in a spatio-temporal model, but also how to do it in a model that includes spatial random effects at two-level of spatial aggregation and space-time interactions at both first or second-level area (census sectors and communes in our data of analysis).

The models are fitted using the recently derived INLA estimation technique, reducing the computational time in comparison with models fitted using MCMC based simulation techniques. The model complexity requires a great amount of time using MCMC while INLA offers a faster alternative, which it could be applied to programs of real-time spatiotemporal representation of dengue risk. In addition, the results from the models applied in the present report could also be used in combination with other statistical methods of spatial and temporal risk representation. For example, space-time clustering methods have been also applied to dengue data by Fuentes-Vallejo [44] in a hyperendemic colombian city located at the center of the country, concluding that there were not specific areas in the city driving the transmission of dengue disease, a characteristic displayed in our results.

As limitations of the study, we account that the use of notification data can lead to under-representation of those cases that are managed at home, without reporting to the surveillance system [45]. Also, some of the addresses possibly were not correctly geocoded, or not geocoded at all, due to mistakes in filling the notification form. Quantifying the percentage of correctly geocoded cases is difficult, although we keep out of the final data those addresses with inconsistent data. In addition, some of the cases reported as dengue were not confirmed by laboratory, but confirmed by clinical diagnosis, leading to a bias difficult to quantify in our results [45]. Furthermore, a current problem for the epidemiological studies at block, section or sector level in Colombia is the lack of updated data from the official statistics, leading to an additional source of bias on the results. Although we report these limitations, we also address that dengue is a highly recognized disease within the medical staff (physicians, nurses, public health personal) in Colombia. Currently, health authorities at city level employ the guidelines [46] published by the Colombian Ministry of Health, the Colombia’s National Health Institute, and the Pan American Health Organization to design and implement activities of entomological surveillance and control of dengue transmission. The guidelines define methodological approaches to spatial mapping of dengue cases and vector data mainly based on descriptive statistics. The epidemiological and statistical tools like the relative risk models shown in this study can help to decrease the dengue burden by providing risk maps and risk profiles, which in first stages will approximate the unknown field situation, and in second stages, with the addition of high quality data, will support an integrated approach to dengue surveillance and control activities [47].

We finally think that further work is needed to make available to the public health policy-makers epidemiological tools to generate real-time dengue disease incidence risk maps, including environmental risk factors (rainfall, humidity, temperature, …) or other potential explanatory variables such as vectorial ecology in the modeling process.
